# Expression and Site-Specific Biotinylation of Human Cytosolic 5′-Nucleotidase 1A in *Escherichia coli*

**DOI:** 10.3390/mps8030066

**Published:** 2025-06-18

**Authors:** Nataliya Slater, Anuradha Sooda, Frank L. Mastaglia, Sue Fletcher, Mark Watson, Merrilee Needham, Jerome D. Coudert

**Affiliations:** 1 Personalised Medicine Center, Murdoch University, Murdoch, WA 6150, Australia; a.sooda@murdoch.edu.au (A.S.); s.fletcher@murdoch.edu.au (S.F.); mark.watson@murdoch.edu.au (M.W.); merrilee.needham@health.wa.gov.au (M.N.); jerome.coudert@univ-tlse3.fr (J.D.C.); 2 Perron Institute for Neurological and Translational Science, University of Western Australia, Nedlands, WA 6009, Australia; francis.mastaglia@perron.uwa.edu.au; 3 Centre for Neuromuscular & Neurological Disorders, University of Western Australia, Crawley, WA 6009, Australia; 4 School of Medicine, University of Notre Dame Australia, Fremantle, WA 6160, Australia; 5 Department of Neurology, Fiona Stanley Hospital, Murdoch, WA 6150, Australia

**Keywords:** cytosolic 5′-nucleotidase 1A, cN1A, AviTag, IBM, autoantibodies, B cells

## Abstract

Autoantibodies targeting cytosolic 5′-nucleotidase 1A (cN1A) are found in several autoimmune diseases, including inclusion body myositis (IBM), Sjögren’s syndrome, and systemic lupus erythematosus. While they have diagnostic relevance for IBM, little is known about the autoreactive B cells that produce these antibodies. To address this, we developed a robust protocol for the expression and site-specific biotinylation of recombinant human cN1A in *Escherichia coli*. The resulting antigen is suitable for generating double-labelled fluorescent baits for the isolation and characterisation of cN1A-specific B cells by flow cytometry. Site-specific biotinylation was achieved using the AviTag and BirA ligase, preserving the protein’s structure and immunoreactivity. Western blot analysis confirmed that the biotinylated cN1A was recognised by both human and rabbit anti-cN1A antibodies. Compared to conventional chemical biotinylation, this strategy minimises structural alterations that may affect antigen recognition. This approach provides a reliable method for producing biotinylated antigens for use in immunological assays. While demonstrated here for cN1A, the protocol can be adapted for other autoantigens to support studies of antigen-specific B cells in autoimmune diseases.

## 1. Introduction

Autoimmune diseases are characterised by aberrant immune responses directed against self-antigens, leading to chronic inflammation and tissue damage. A central feature of many autoimmune conditions is the presence of antibodies produced by autoreactive B cells, which not only serve as disease biomarkers but may also directly contribute to pathogenesis [1]. Understanding the specificity and functional characteristics of these autoreactive B cell populations is essential for elucidating disease mechanisms and informing the development of sensitive diagnostic assays and targeted therapies.

In humans, the proportion of B cells specific to a given antigen typically comprises less than 0.5% of circulating B cells, even at the peak of post-vaccination expansion [2]. Consequently, characterisation of these rare populations is challenging and requires an enrichment step. Among the available strategies, fluorescence-activated cell sorting (FACS) using mono- or multimeric antigen probes has emerged as a powerful approach. Initially developed for the isolation of antigen-specific T cells using MHC-peptide tetramers [3], this technology was later adapted for B cells with the introduction of antigen tetramers [4]. These probes are typically generated by conjugating biotinylated antigens to fluorochrome-labelled streptavidin, producing multivalent complexes that bind B cell receptors (BCRs) with enhanced avidity. In certain contexts, however, monomeric antigens have proven sufficient for the successful isolation of antigen-specific B cells [5].

Owing to its exceptional affinity and stability, the biotin–streptavidin coupling system is ideally suited for such applications. Biotinylation using N-hydroxysuccinimide (NHS) chemistry enables the covalent attachment of biotin to primary amine groups, typically located on lysine side chains and the protein’s N-terminus. However, this non-specific approach may inadvertently modify lysine residues critical for proper protein folding or the preservation of epitope integrity, potentially affecting the probe specificity. To overcome this limitation, site-specific enzymatic biotinylation methods—such as the approach developed by Chen and colleagues—employ a short AviTag sequence that is specifically recognised by the *E. coli* biotin ligase BirA [6]. This strategy ensures a consistent and structurally defined biotin modification, which is critical for maintaining immunogenic and conformational epitopes.

The 5′-nucleotidases (5NTs) are a family of enzymes that catalyse the dephosphorylation of nucleoside monophosphates to produce nucleosides and inorganic phosphate, thereby regulating intracellular nucleotide pools. Dysregulation of intracellular 5′-nucleotidases has been implicated in a range of human diseases. Notably, autoantibodies directed against cytosolic 5′-nucleotidase 1A (cN1A) have been identified in patients with inclusion body myositis (IBM), Sjögren’s syndrome, systemic lupus erythematosus, and multiple sclerosis [7,8,9,10]. Emerging evidence suggests that the presence of these autoantibodies may be associated with more severe disease phenotypes [11,12,13], although their precise role in pathogenesis remains to be fully elucidated [14,15]. The ability to identify and characterise cN1A-reactive B cells using fluorescently labelled antigenic probes offers a promising avenue to investigate the structure and function of these autoantibodies. Such studies could enhance our mechanistic understanding of disease processes and inform the development of novel targeted immunotherapies.

Recombinant cN1A with a C-terminal hexa-histidine tag has previously been expressed in *Escherichia coli* RosettaBlue(DE3)pLacI and employed in the development of a diagnostic ELISA for anti-cN1A autoantibody detection [16]. In the present study, we describe the expression of C-terminally hexa-histidine tagged cN1A in *E. coli* BL21(DE3)pLysS, followed by site-specific biotinylation using BirA ligase.

## 2. Materials and Methods

General Laboratory equipment and consumables required are listed below. We do not recommend any particular brand.

- Steam steriliser;
- Incubator shaker;
- Benchtop centrifuge;
- Benchtop microcentrifuge;
- Refrigerated high-speed centrifuge;
- Sonicator;
- Microplate spectrophotometer;
- pH meter;
- Magnetic stirrer and stir bars;
- Gel casting equipment;
- Gel electrophoresis system;
- Refrigerators and freezers (4 °C, −20 °C, and −80 °C);
- Flow cytometer;
- Air-displacement pipettes and tips;
- Pipette controller and serological pipettes;
- Centrifuge tubes (1.5 mL, 15 mL, and 50 mL);
- 10 cm Petri dishes;
- Baffled shake flasks (250 mL, 500 mL, and 1000 mL);
- Disposable sterile inoculating loops;
- Orbital shaker;
- Rolling platform;
- Gel imaging system;
- 10 mL syringes;
- 18 gauge needles;
- 2 mL screw-top tubes;
- Trypan Blue solution 0.4%;
- Cell counting slides.

The kits and equipment used in the experiments are detailed in Table 1, and the chemical reagents required are summarised in Table 2. Buffer and bacterial growth media compositions are provided in Table 3.

### 2.1. cN1A Expression Vector

**Figure 1:**
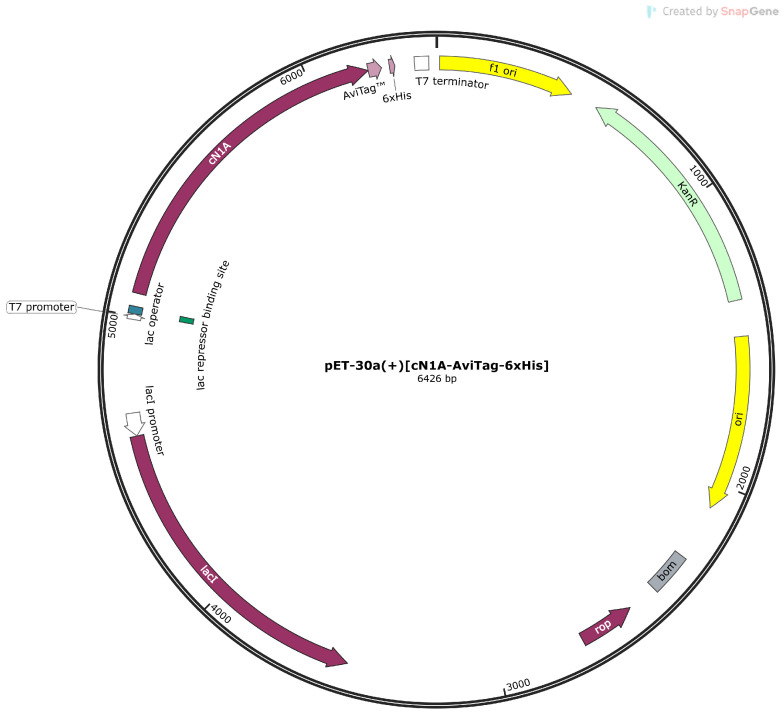
Circular map of the recombinant cN1A-AviTag inserted into pET-30a(+) expression vector. The arrows indicate the location of a gene or feature on the plasmid and the direction of transcription. The map was produced by SnapGene Viewer version 7.0.1 (GSL Biotech LLC, Chicago, IL, USA).

Complementary DNA (cDNA) encoding the 368-amino acid sequence of human cN1A (UniProt accession number Q9BXI3) with *E. coli*-optimised codons (Supplementary Data S1) and inserted into a pET30a(+) prokaryotic expression vector was purchased from GenScript Biotech (Piscataway, NJ, USA). The plasmid map, with relevant annotated features, is shown in Figure 1. Notable features comprise an AviTag sequence followed by a hexa-histidine tag at the C-terminus. The 15-amino acid AviTag sequence—GLNDIFEAQKIEWHE—is recognised by the *E. coli* enzyme BirA that attaches a single biotin molecule to the lysine residue of the sequence [17], enabling targeted biotinylation of the protein. The plasmid expression is regulated by a T7 promoter, which is typically suppressed by the lac repressor bound to the lac operator site. The repressor is displaced in the presence of lactose or isopropyl β-D-1-thiogalactopyranoside (IPTG), a synthetic analogue of allolactose. The vector also contains a kanamycin resistance gene for the selection of transformed bacterial hosts.

### 2.2. E. coli Host Strain

BL21(DE3)pLysS *E.coli* strain kindly donated by Assoc Prof Mark Watson was used for protein expression. This strain’s genotype and critical features are summarised in Table 4. The absence of the Lon and OmpT proteases is important for reducing unwanted protein degradation during expression. The λDE3 region harbours the *lacI* gene, which encodes the lac repressor, and the lacUV5-T7p07promoter, which is used to control the expression of T7 RNA polymerase. This system enables tight regulation of recombinant protein expression as the T7 polymerase is only produced upon induction with IPTG, allowing for controlled transcription of the gene of interest. The pLysS plasmid provides an additional layer of regulation, containing the *CmR* chloramphenicol resistance gene and T7 phage lysozyme, which reduces leaky expression of the recombinant protein when not induced, helping to mitigate potential stress on the bacterial system.

### 2.3. Electrocompetent E. coli Preparation

*E. coli* cells are generally inefficient at taking up exogenous DNA under natural conditions [18]. However, their competency can be significantly enhanced through artificial means such as exposure to elevated Ca^+^ concentrations, heat shock, polyethylene glycol treatment, or electroporation, as employed in this protocol [18].

To prepare a high-density suspension of electrocompetent *E. coli*:

1. Streak BL21(DE3)pLysS *E. coli* onto an LB agar plate and incubate overnight at 37 °C.
2. Select a single colony and inoculate it into 30 mL of SOB medium.
3. Incubate overnight at 37 °C with shaking at 200 RPM.
4. Inoculate 1 L SOB medium with 10 mL of the overnight culture and incubate at 37 °C with shaking at 200 RPM.
5. Monitor bacterial growth by transferring 100 µL of the culture into a 96-well flat-bottom plate every 30–60 min and measuring optical density at 600 nm (OD_600_) using a microplate reader.
6. Continue incubation for approximately 4 h until OD_600_ reaches 0.5.
7. Transfer the culture into 10 × 50 mL centrifuge tubes using a 25 mL serological pipette.
8. Store the remaining culture at 4 °C until ready to process.
9. Centrifuge the tubes at 1700× *g* for 10 min at 4 °C to pellet the cells.
10. Discard the supernatant.
11. Fill the tubes with the remaining culture and repeat centrifugation.
12. Discard the supernatant.
13. Wash the pellets three times with 40 mL cold 10% glycerol by resuspending the cells and centrifuging at 1700× *g* for 10 min at 4 °C.
14. Resuspend the final pellets in 5 mL cold 10% glycerol and combine all cells into two 50 mL centrifuge tubes.
15. Centrifuge again at 1700× *g* for 10 min at 4 °C and discard the supernatant.
16. Resuspend each pellet in 750 µL cold 10% glycerol.
17. Aliquot 100 µL into 2 mL screw-cap tubes and store at −80 °C as electrocompetent glycerol stocks.

### 2.4. Transformation of Electrocompetent E. coli with the cN1A Vector

1. Thaw100 µL of electrocompetent glycerol stock on ice.
2. Divide the thawed cells evenly into two 1.5 mL microcentrifuge tubes:
  - One tube for transformation (T).
  - One tube as an untransformed control (C).
3. Add 100 pg of pET-30a(+)[cN1A] plasmid DNA to the T tube and gently mix by flicking.
4. Add the same volume of sterile water to the C tube and gently mix by flicking.
5. Incubate both tubes on ice for 10–15 min.
6. Transfer the transformation mixture into a pre-chilled electroporation cuvette.
7. Perform electroporation using the following settings:
  - Voltage: 1800 V
  - Pulse duration: 3.8 s
8. Immediately add 1950 µL of SOC medium to the cuvette.
9. Transfer the contents into a 15 mL centrifuge tube.
10. Incubate at 37 °C with shaking at 200 RPM for 1 h to allow recovery.
  Important: Loosely cap the tube to allow aeration.
11. After recovery, add each entire culture to 5 mL of LB medium in separate 50 mL centrifuge tubes and incubate overnight at 37 °C with shaking at 200 RPM.
  Important: Loosely cap the tubes to allow aeration.
12. Treat the control tube identically, except for the following:
  - Supplement the LB medium with 34 µg/mL chloramphenicol only (no kanamycin).
13. The next day, assess both cultures for turbidity as an indication of bacterial growth.
14. Centrifuge the transformed culture at 3800× *g* for 10 min at 8 °C.
15. Discard the supernatant and resuspend the cell pellet in 500 µL of cold 10% glycerol.
16. Transfer the entire volume into a 2 mL screw-cap tube and store at −80 °C as transformed glycerol stock.

### 2.5. Small-Scale Expression Analysis

The purpose of the small-scale expression analysis is to confirm inducible expression of the target protein and to determine its subcellular localisation.

1. Using a sterile inoculation loop, gently touch the surface of the transformed *E. coli* BL21(DE3)pLysS[cN1A] glycerol stock.
  Important: Do not allow the stock to thaw.
2. Streak the bacteria onto an LB agar plate.
3. Incubate the plate overnight at 37 °C.
4. Using a sterile inoculation loop, transfer a single colony into 3 mL of LB medium in a 50 mL centrifuge tube.
5. Incubate at 37 °C with shaking at 200 RPM for 3 h.
  Important: Loosely cap the tubes to allow aeration.
6. Transfer the entire 3 mL starter culture into 100 mL of LB medium in a 500 mL baffled shaker flask.
7. Incubate at 37 °C with shaking at 200 RPM.
8. Monitor culture growth hourly by measuring OD_600_.
9. When OD_600_ reaches 0.6, divide the culture evenly into two 50 mL subcultures in 250 mL baffled shaker flasks.
10. Induce one subculture with 1 mM IPTG; leave the second as an uninduced control.
11. Continue incubation for 4 h at 37 °C with shaking at 200 RPM.
12. Collect 1 mL from each culture into two 1.5 mL microcentrifuge tubes for total protein analysis.
13. Centrifuge at 10,000× *g* for 3 min to pellet the cells.
14. Discard the supernatant.
15. Resuspend each pellet in 100 µL PBS and add 100 µL of 2× sample loading buffer.
16. Freeze samples at −20 °C until SDS-PAGE analysis.
17. Centrifuge remaining cultures at 6000× *g* for 10 min to pellet the cells.
18. Transfer the supernatant to fresh 50 mL centrifuge tubes.
19. To analyse the extracellular fraction, transfer 0.5 mL of the supernatant to a 10 kDa molecular weight cut-off (MWCO) centrifugal filter unit.
20. Centrifuge at 5000× *g* for 20 min (until concentrated to 100 µL) at 8 °C.
21. Mix the concentrate with an equal volume of 2× sample loading buffer.
22. Store at −20 °C until SDS-PAGE.
23. Resuspend cell pellets in 4 mL cold cell lysis buffer.
24. Sonicate using 10 bursts of 5 s each at 40% amplitude.
25. Centrifuge the lysate at 14,000× *g* for 10 min to separate soluble and insoluble material.
26. Transfer the supernatant containing the soluble fractions to fresh 15 mL centrifuge tubes.
27. Mix 100 µL of the soluble fractions with 2× sample loading buffer and freeze at −20 °C until SDS-PAGE analysis.
28. Wash the insoluble pellet (inclusion bodies) twice with 750 µL PBS:
  (a) Resuspend the pellet thoroughly.
  (b) Centrifuge at 10,000× *g* for 10 min.
  (c) Discard the supernatant.
29. Resuspend the final pellet in 1.5 mL of urea denaturing buffer.
30. Alternate vortexing and incubating for 10–15 min until the pellet is fully solubilised.
31. Mix 100 µL of this fraction with 2× sample loading buffer.
32. Freeze at −20 °C until SDS-PAGE.

### 2.6. Optimisation of Induction Conditions Using Dot Blot Assay

Dot blotting is a rapid and straightforward immunoassay technique used to detect specific proteins or epitopes within a complex mixture, without requiring electrophoretic separation. In this study, the dot blot assay is employed to optimise induction conditions for the recombinant protein expression, including the point of induction, induction duration and temperature, and the concentration of the inducer.

The induction conditions tested in this study are summarised in Table 5.

1. Prepare samples for analysis.
  (a) Prepare a small-scale culture as described in steps 1–8 of Section 2.5.
  (b) Monitor OD_600_ regularly. When OD_600_ reaches 0.6, aliquot 10 mL of culture into each of five 50 mL centrifuge tubes and label as test conditions 1, 3, 4, 7, and 8.
  (c) Immediately add IPTG as follows:
    - 0.5 mM IPTG to tubes 1 and 7.
    - 1.0 mM IPTG to tubes 4 and 8.
    - Do not add IPTG to tube 3 (uninduced control).
  (d) Incubate the tubes under the conditions listed in Table 5:
    - Tubes 1, 3, and 4: 16 h at 23 °C.
    - Tubes 7 and 8: 4 h at 37 °C.
  (e) Continue incubating the remaining culture until OD_600_ reaches 1.0.
  (f) Aliquot 10 mL of culture into each of five additional 50 mL centrifuge tubes and label as test conditions 2, 5, 6, 9, and 10.
  (g) Add IPTG as follows:
    - 0.5 mM IPTG to tubes 2 and 5.
    - 1.0 mM IPTG to tubes 6 and 10.
    - Do not add IPTG to tube 9 (uninduced control).
  (h) Incubate these tubes under the following conditions:
    - Tubes 2 and 6: 16 h at 23 °C.
    - Tubes 5, 9, and 10: 4 h at 37 °C.
  (i) At the end of the induction period, transfer 100 µL aliquots from each culture into 1.5 mL microcentrifuge tubes.
  (j) Lyse the cells by sonicating at 20 kHz using ten consecutive 1 s bursts.
  (k) Store the lysates at −20 °C until the analysis.
2. Perform dot blot analysis:
  (a) Spot 5 µL of each lysate directly onto a nitrocellulose membrane.
  (b) Label the membrane with a permanent marker indicating the position and identity of each spot.
  (c) Allow the membrane to air-dry at room temperature for 20 min.
  (d) Block unoccupied binding sites by incubating the membrane in 5% skimmed milk/0.1% Tween-20/TBS buffer (blocking buffer) for 1 h at 23 °C on an orbital shaker.
  (e) Prepare 10 mL of rabbit anti-cN1A primary antibody diluted 1:500 in blocking buffer. Flood the membrane and incubate for 1 h at 23 °C on a rolling platform.
  (f) Recover the primary antibody solution and store at −20 °C.
    Note: This solution may be reused up to three times without loss of efficacy.
  (g) Wash the membrane three times in 20 mL 0.1% Tween-20/TBS (TBST) for 10 min each wash at 23 °C on an orbital shaker.
  (h) Prepare 10 mL of goat anti-rabbit IgG-HRP secondary antibody diluted 1:10,000 in blocking buffer. Flood the membrane and incubate for 1 h at 23 °C on a rolling platform.
  (i) Recover the secondary antibody solution and store at −20 °C.
    Note: This solution may also be reused up to three times without loss of efficacy.
  (j) Repeat the washing steps. During the final wash, replace TBST with TBS.
  (k) Prepare 7 mL of enhanced chemiluminescence (ECL) solution according to the manufacturer’s instructions.
  (l) Incubate the membrane in ECL substrate for 10 min.
  (m) Visualise and quantify the chemiluminescent signal using a gel imaging system and software.

### 2.7. Large-Scale Protein Expression

1. Prepare a starter culture as described in Steps 1–5 of Section 2.5.
2. Inoculate 250 mL of LB medium in a 1 L baffled flask with the entire 3 mL starter culture.
3. Incubate the culture for 2 h at 37 °C with shaking at 200 RPM.
4. Induce protein expression by adding 125 µL of IPTG (final concentration 0.5 mM). Continue incubation at 37 °C, 200 RPM for 4 hs.
5. Harvest the culture in five 50 mL conical tubes by centrifugation at 6000× *g*, 8 °C for 10 min.
6. Wash each pellet twice with 40 mL of cold PBS.
7. Resuspend the pellets in 5 mL of cold PBS, and pool all pellets into a single 50 mL conical tube.
8. Centrifuge one more time at 6000× *g* at 8 °C for 10 min, and discard the supernatant.
9. Either store the final washed pellet at −20 °C or proceed to lysis:
  (a) Resuspend the pellet in 5 mL cold lysis buffer.
  (b) Lyse cells by sonication (5 cycles of 10 s on / 10 s off at 40% amplitude).
10. Centrifuge the lysate at 6000× *g* at 8 °C for 10 min. Collect the supernatant into a fresh 15 mL conical tube (soluble fraction).
11. Inspect the pellet. If uneven in colour (light and dark patches), repeat the lysis step with an additional 1–2 mL of lysis buffer.
12. Confirm successful enrichment of inclusion bodies by the presence of a homogeneously pale, sand-coloured pellet.
13. Resuspend the final pellet in 2 mL of urea denaturing buffer.
14. Alternate vortexing and incubating for 10–15 min until the pellet is fully solubilised.
15. Proceed immediately to protein purification or store the lysate at −20 °C.

### 2.8. Protein Purification

Recombinant cN1A is purified from soluble (lysate) and insoluble (inclusion bodies) fractions under denaturing conditions using a Qiagen Fast Start Ni-NTA column and buffers supplied as a kit.

1. Prepare buffers:
  (a) Adjust wash buffer and elusion buffer pH with concentrated HCl.
  (b) Adjust elution buffer pH with concentrated NaOH.
2. Prepare the Ni-NTA column:
  (a) Gently resuspend the resin in a Fast Start column by inversion several times.
  (b) Break the seal at the outlet of the column.
  (c) Open the screw cap and allow the storage buffer to drain out.
    Important: The outlet seal must be broken before the screw cap is removed.
3. Bind the His-tagged protein:
  (a) Apply up to 10 mL of the clear cell lysate supernatant to the column.
  (b) Allow the lysate to flow through the column resin under gravity.
  (c) Collect the flow-through fraction into a 15 mL centrifuge tube.
  (d) Load the same column with up to 10 mL of the lysed inclusion bodies.
  (e) Collect the flow-through fraction into a new 15 mL centrifuge tube.
  (f) Add 5 µL of 2× sample loading buffer to 5 µL of the flow-through from each fraction and store at −20 °C for SDS-PAGE analysis.
4. Wash the column:
  (a) Wash the column twice with 4 mL of denaturing wash buffer.
  (b) Collect each wash fraction into a separate 15 mL centrifuge tube.
  (c) Add 5 µL of 2× sample loading buffer to 5 µL of each wash and store at −20 °C for SDS-PAGE analysis.
5. Elute the His-tagged protein:
  (a) Elute bound protein twice with 1 mL of denaturing elution buffer.
  (b) Collect each eluted fraction into a separate 15 mL centrifuge tube.
  (c) Add 5 µL of 2× sample loading buffer to 5 µL of each wash and store at −20 °C for SDS-PAGE analysis.
6. Dialyse the eluted protein for biotinylation:
  (a) Hydrate a Slide-A-Lyzer dialysis cassette with a 3500 Da MWCO by submerging it into a deionised water for 2 min.
  (b) Using an 18-gauge needle, inject up to 10 mL of the eluted cN1A into the cassette.
    Note: Mark the corner used for injecting the sample with a permanent marker.
  (c) Secure the cassette in a float buoy ensuring that the injecting site is at the top.
    Warning: While the cassette has an internal gasket that seals after the needle is withdrawn, its failure may result in the loss of protein unless the cassette is secured the correct way up.
  (d) Float the cassette in 1 L of 50 mM bicine buffer (pH 8.3).
  (e) Dialyse overnight at 4 °C with gentle stirring.
7. Analyse purification fractions:
  (a) Analyse the flow-through, wash, and elution fractions with SDS-PAGE and Western blot as described in Section 2.11.

### 2.9. Determination of Protein Concentration by Bicinchoninic Acid (BCA) Assay

Recombinant cN1A-AviTag concentration is determined using Pierce BCA protein assay kit following the manufacturer’s protocol for a microplate procedure with volume adjustments.

1. Prepare working reagent (WR) by mixing BCA Reagent A and BCA Reagent B at a 50:1 ratio (2 mL A + 40 µL B is sufficient for the set of standards and one unknown sample).
2. Prepare a dilution series of bovine serum albumin (BSA) in the same buffer as the samples, following the volumes outlined in Table 6.
3. Pipette 25 µL of each standard and undiluted cN1A-AviTag into a 96-well flat-bottom plate in duplicate.
4. Add 200 µL of WR to each well using a multichannel pipette.
5. Mix the samples thoroughly by pipetting three times.
6. Seal the plate with a plastic cover and incubate at 37 °C for 30 min.
  Note: Incubation time can be increased to lower the minimum detection level of the reagent and the working range of the assay as long as the standards and the samples are treated identically.
7. Allow the plate to cool to 23 °C (approximately 10 min).
8. Measure the absorbance at 562 nm using a microplate reader.
9. Generate a standard curve from the BSA standards and calculate the concentration of the unknown sample using the best-fit line.

### 2.10. Recombinant cN1A Biotinylation

cN1A biotinylation is performed using BirA500RT kit (Avidity LLC, Aurora, CO, USA USA) according to the manufacturer’s protocol.

1. Reconstitute the lyophilised BirA ligase in 20 µL of the BirA Resuspension buffer (both supplied).
2. Add ATP to the 10× SuperMix buffer (both supplied).
3. Prepare a 4 mL biotinylation reaction by mixing:
  - 400 µL of 10× SuperMix.
  - 8 µg of BirA ligase.
  - 1.8 mg of cN1A-AviTag.
4. Incubate the reaction mixture for 35 min at RT with gentle agitation.
5. Dialyse the biotinylated protein to remove unreacted biotin and exchange the reaction buffer for storage buffer (50 mM Tris, 500 mM NaCl, pH 8.0). Follow the procedure described in Section 2.8 Step 5.
6. Determine the concentration of biotinylated cN1A using the BCA assay, as described in Section 2.9.

### 2.11. Gel Electrophoresis and Western Blot

Sodium dodecyl sulphate-polyacrylamide gel electrophoresis (SDS-PAGE) is carried out using a hand-casted 4–12% polyacrylamide gel.

1. SDS-PAGE:
  (a) Prepare the resolving 12% polyacrylamide gel solution:
**Component****Final Concentration / Volume**40% Acrylamide Solution (29:1 bis-acrylamide ratio)12%1.5 M Tris-HCl, pH 8.825%diH_2_O4.4 mLSDS ^1^0.1%APS ^2^0.05%TEMED ^3^0.05%^1^ Prepare as a 20% stock solution and store at room temperature. ^2^ Prepare as a 10% stock solution, aliquot, and store at −20 °C. ^3^ Add immediately before pouring the gel.
  (b) Assemble the casting module using 1 mm glass plates.
  (c) Pour the resolving gel solution to fill approximately three-quarters of the cassette height.
  (d) Carefully overlay the gel with 1 cm of deionised water using a transfer pipette to prevent air bubbles entering the gel and to level the gel surface.
  (e) Allow the gel to polymerise at ambient temperature for 20 min.
  (f) Meanwhile, prepare the stacking 4% polyacrylamide gel solution:
**Component****Final Concentration/Volume**40% Acrylamide Solution (29:1 bis-acrylamide ratio)4%0.5 M Tris-HCl, pH 6.825%Glycerol25%Bromophenol blue0.0048%SDS ^1^0.1%APS ^2^0.05%TEMED ^3^0.05%^1^ Prepare as a 20% stock solution and store at room temperature. ^2^ Prepare as a 10% stock solution, aliquot, and store at −20 °C. ^3^ Add immediately before pouring the gel.
  (g) Pour off the water from the polymerised resolving gel.
  (h) Pour the stacking gel solution on top of the resolving gel.
  (i) Carefully insert a 10-well 1 mm comb without trapping air bubbles.
  (j) Allow the stacking gel to polymerise for 20 min at ambient temperature.
  (k) Thaw previously prepared samples and heat for 10 min at 95 °C to denature proteins.
  (l) Assemble the gel tank and fill it with SDS-PAGE running buffer.
  (m) Gently remove the comb from the gel and rinse the wells with SDS-PAGE running buffer using a transfer pipette.
  (n) Load each well with 10 µL of sample or 5 µL of Precision Plus protein standard (Bio-Rad).
    Note: Load empty wells with 1× sample loading buffer.
  (o) Perform electrophoresis at 17 V/cm (equivalent to 120 V in a Bio-Rad Mini-PROTEAN tank) for 90 min.
    Warning: Always turn off the power before opening the electrophoresis tank.
2. Coomassie blue staining:
  Warning: Do not perform Coomassie blue staining if protein transfer is required for Western blotting.
  (a) Remove the gel from the glass cassette into a suitable staining container.
  (b) Submerge the gel in 0.1% Coomassie blue solution for 10 min on an orbital shaker.
  (c) Discard the staining solution and destain the gel in deionised water overnight on an orbital shaker.
3. Western blotting:
  (a) Remove the gel from the glass cassette and assemble the transfer cassette by placing the SDS-PAGE gel and a nitrocellulose membrane between filter papers saturated with 1× transfer buffer.
    Note: Pre-assembled transfer sandwiches are pre-saturated with transfer buffer.
  (b) Perform semi-dry protein transfer at 1.3 A for 7 min.
    Note: If using the Power Blotter system (Invitrogen), select the pre-set Mixed-Range MW (25–150 kDa) programme.
  (c) Block the membrane with 5% skimmed milk/TBST blocking buffer for 1 h at 23 °C.
  (d) Incubate with primary antibody overnight at 4 °C on a rolling platform, using the dilutions listed in Table 7.
  (e) Wash the membrane three times for 10 min each in TBST on a rotary shaker.
  (f) Incubate with secondary antibody for 1 h at room temperature on a rolling platform, using the dilutions listed in Table 7.
  (g) Wash the membrane two times for 10 min each in TBST, followed by a final 10 min wash in TBS.
  (h) For chemiluminescent signal development, submerge the membrane in 7 mL ECL substrate for 10 min at ambient temperature.
  (i) Image the bands using a gel imaging system.

### 2.12. Example of Application: Identification of cN1A-Reactive B Cells by Flow Cytometry

Warning: All human blood samples must be treated as potentially infectious. Personal protective equipment must be worn when handling blood or blood-derived materials. All procedures should be conducted in a certified biosafety cabinet, and waste must be disposed of in accordance with institutional biosafety regulations.

1. Isolate Peripheral Blood Mononuclear Cells (PBMCs) from 40 to 50 mL of venous blood using your laboratory’s preferred protocol.
  Note: Blood should be processed within two hours of collection to ensure high cell viability.
2. Count the cells using the Trypan Blue exclusion method and manual or automated counting.
3. Resuspend PBMC at a concentration of 5 × 10^7^ cells/mL in PBS containing 2% foetal calf serum (FCS) and 1 mM EDTA for B cell enrichment.
  Alternatively, PBMC may be cryopreserved for analysis at a later timepoint.
4. Enrich B cells using the EasySep Human Pan-B Cell Enrichment Kit (STEMCELL Technologies) following the manufacturer’s instructions.
5. Count the enriched B cells using the Trypan Blue exclusion method and manual or automated counting.
6. Resuspend B cells in RPMI 1640 supplemented with 5% FCS at a concentration of 1 × 10^6^ cells/mL and transfer into a well of an appropriate culture plate based on the final suspension volume.
7. Add recombinant biotinylated cN1A at 10 μg/mL and incubate the plate at 37 °C with 5% CO_2_ for one hour to allow cN1A to bind.
8. Collect the cells and transfer into a 5 mL round-bottom tube.
9. Centrifuge the suspension at 400× *g* for 5 min to pellet the cells and discard the supernatant.
10. Wash the cells by resuspending the pellet in 2 mL PBS with 2% FCS (FACS buffer) and then centrifuge again at 400× *g* for 5 min to remove unbound cN1A.
11. Prepare 100 μL of staining cocktail per 1 × 10^6^ cells by adding fluorochrome-conjugated reagents (Table 8) to FACS buffer.
12. Add the staining cocktail to the cells, gently resuspend the pellet, and stain in the dark on wet ice for 20 min.
13. Wash the cells with 2 mL FACS buffer.
14. Resuspend the pellet in 300 μL FACS buffer and acquire on a flow cytometer.
15. Gate on live (EF506^−^) CD19^+^ BV421^+^ CF488^+^ cells to identify cN1A-reactive B cells.

## 3. Results

### 3.1. Analysis of Intracellular and Extracellular cN1A Expression

**Figure 2:**
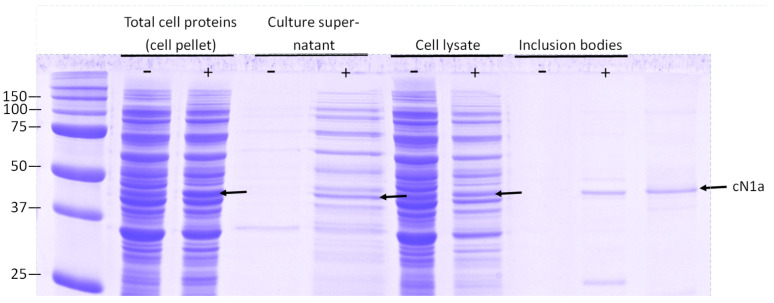
SDS-PAGE analysis of proteins obtained from various *E. coli* culture fractions. Bacterial culture was induced with 1 mM IPTG for 4 h at 37 °C (labelled with “+”). Control culture was grown under the same conditions without the addition of IPTG (samples labelled with “−”). Arrows indicate the band corresponding to cN1A.

Samples from various fractions of the bacterial culture post-induction were simultaneously analysed by SDS-PAGE (Figure 2). Recombinant cN1A was detected in the bacterial lysate and in the denatured inclusion bodies. Additionally, a small amount of the protein was present in the culture supernatant. As the expression vector lacked any secretion signals, active transport of cN1A into the extracellular space was unlikely. The protein leakage likely resulted from bacterial lysis induced by metabolic stress associated with recombinant protein expression. This was consistent with the observed reduction in cell density in the induced culture (Figure 2) and the presence of other bacterial proteins in the supernatant.

### 3.2. Induction Condition Optimisation

**Figure 3:**
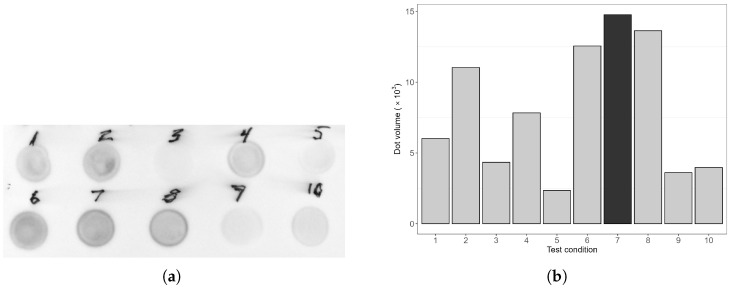
Optimisation of protein expression induction using a dot blot assay. (**a**) Dot blot image showing protein expression under various induction conditions, as numbered and detailed in Table 5. Tested variables include time of induction, duration, temperature, and IPTG concentration. (**b**) Quantitative analysis of dot intensities; larger dot volumes correspond to higher protein concentrations. Orange colour indicates the set of conditions yielding the highest protein expression. Analysis was performed with Vilber Fusion FX software (Vilber Lourmat, Eberhardzell, Germany).

The yield of recombinant cN1A protein was strongly influenced by the induction conditions. Variations in dot intensity observed in Figure 3 reflect corresponding differences in the concentration of cN1A in the *E.coli* lysate following induction. The optimal yield was achieved by inducing cultures when OD_600_ reached 0.6 with 0.5 mM IPTG, followed by incubation for 4 h at 37 °C. Notably, expression of cN1A was also detected in uninduced controls (Figure 3a, dots 3 and 9), likely due to basal or “leaky” expression from the plasmid in absence of the inducer or as a native bacterial cN1A.

### 3.3. Monitoring of the Protein Purification

**Figure 4:**
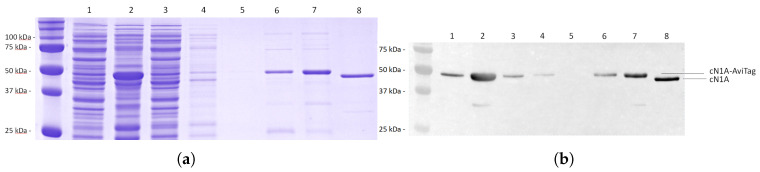
Purification of cN1A-AviTag-His-Tag using a Qiagen Ni-NTA column. Lanes: 1 bacterial lysate, 2 solubilised inclusion bodies, 3 column flow-through, 4 first wash, 5 second wash, 6 first elution, 7 second elution, 8 recombinant cN1A-HisTag (GenScript, Piscataway, NJ, USA). (**a**) SDS-PAGE analysis. (**b**) Western blot probed with human anti-cN1A antibodies and goat anti-human IgG-HRP.

Monitoring each step of the protein purification process (Figure 4) indicated some loss of recombinant protein during the initial binding step (lane 3), likely due to the column oversaturation. Notably, more protein was recovered in the second elution than in the first (lanes 6–7), while a third elution yielded negligible protein (data not shown). The recombinant protein (lanes 1–7) appeared at a higher molecular weight than the commercially produced control (lane 8), consistent with the addition of the AviTag.

### 3.4. AviTag-cN1A Biotinylation

**Figure 5:**
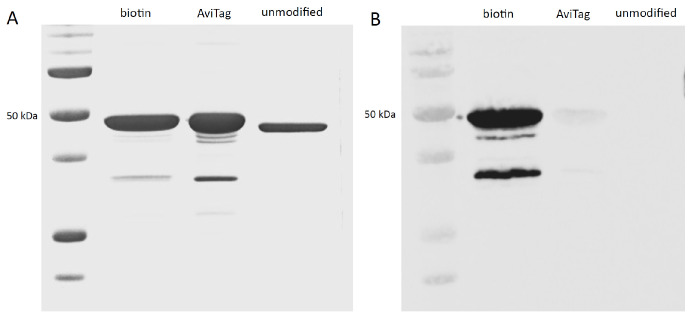
Biotinylation efficiency of AviTag-cN1A by BirA enzyme. (**A**) SDS-PAGE: 5 µL of cN1A protein was loaded per well. Electrophoresis was performed at 120 V for 1 h and 45 min, and the gel was stained with Coomassie Brilliant Blue. (**B**) Western blot: Separated proteins were transferred onto a nitrocellulose membrane and probed with streptavidin-HRP (1:1000 dilution). Image acquisition was performed using the Vilber Fusion FX system.

The entire batch of AviTag-cN1A was subjected to biotinylation. The efficiency of BirA-mediated biotinylation was visually estimated to be close to 100% by comparing the intensity of the protein bands on SDS-PAGE and the corresponding bands on the Western blot probed with streptavidin-HRP (Figure 5).

**Figure 6:**
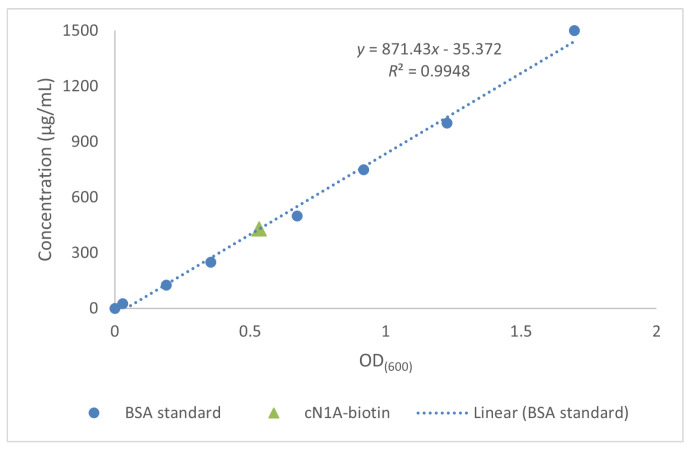
Bovine serum albumin standard curve for the calculation of cN1A-AviTag concentration by bicinchoninic acid assay.

The final concentration of biotinylated cN1A was determined to be 430 µg/mL (Figure 6). This corresponds to a total yield of 2.15 mg of the biotinylated protein obtained from the initial 1 L of bacterial culture.

### 3.5. Antibody Recognition of the Recombinant Biotinylated cN1A

**Figure 7:**
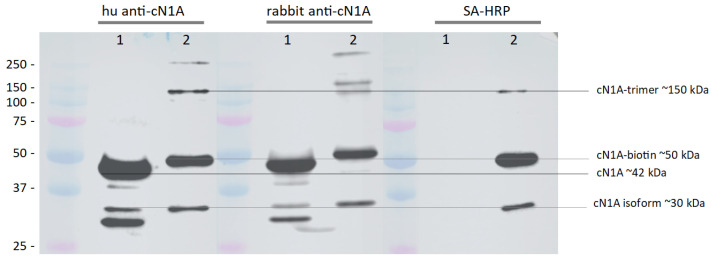
Comparative Western blot analysis of commercially produced recombinant cN1A (1) and in-house expressed and biotinylated cN1A (2). Approximately 1.7 µg of each protein was resolved in triplicate by SDS-PAGE and transferred to a nitrocellulose membrane. The membrane was then sectioned and probed with either α-cN1A antibodies or streptavidin (SA), as outlined in Table 7. Imaging was performed using the Odyssey M imaging system (LI-COR Bio).

Western blot analysis of the in-house expressed and biotinylated cN1A was performed using anti-cN1A antibodies generated in rabbit or purified from sera of human patients, along with HRP-conjugated streptavidin (Figure 7). Commercial recombinant cN1A protein (GenScript, Piscataway, NJ, USA)served as a positive control. Both primary antibodies effectively detected the biotinylated cN1A.

Additional bands were observed for both biotinylated and non-biotinylated cN1A. A band at ≈30 kDa, detected using the anti-cN1A antibodies and streptavidin-HRP, appears specific to cN1A and likely represents a truncated isoform retaining the C-terminal AviTag but lacking part of the N-terminal sequence.

Furthermore, bands corresponding to multimeric forms of biotinylated cN1A were identified. A consistent band at ≈150 kDa, likely representing a trimer of identical 50 kDa subunits, was detected across all three blots. In addition, both human and rabbit anti-cN1A antibodies identified higher molecular weight bands of ≈250 kDa, suggestive of higher-order aggregates.

### 3.6. Verification of Biotinylated cN1A Binding to Circulating B Cells

**Figure 8:**
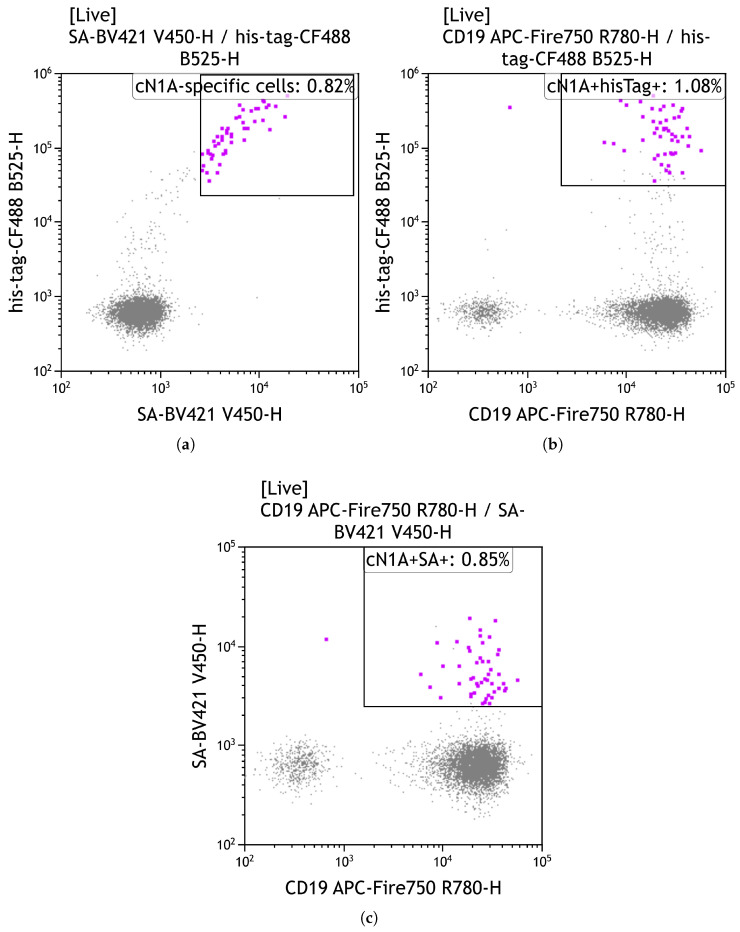
Identification of rare cN1A-specific B cells by flow cytometry. CD19^+^ B cells were enriched from peripheral blood mononuclear cells of a patient with inclusion body myositis and incubated with biotinylated recombinant cN1A for 1 h at 37 °C, followed by staining with a fluorochrome-conjugated antibody cocktail. (**a**) Gating on cN1A-specific cells using dual labelling of polyhistidine and biotin tags to identify cN1A-specific cells; (**b**) Gating using polyhistidine tag only; (**c**) Gating using biotin tag only. Images were generated with Kaluza Analysis v2.3 (Beckman Coulter, Brea, California, USA).

Biotinylated cN1A probes were used to identify circulating cN1A-reactive B cells in IBM patients by flow cytometry (Figure 8). The probes were not intentionally multimerised prior to the assay; however, we were unable to confirm whether they retained a monomeric structure or underwent spontaneous multimerisation.

Cells binding the cN1A probe were detected by BV421 fluorescence via the streptavidin–biotin interaction. To verify probe specificity, an anti-His-tag antibody conjugated to CF488 was used. Double-positive cells (BV421^+^/CF488^+^) were classified as cN1A-reactive (Figure 8). We observed a slight increase in staining specificity when using a combination of anti-His-tag antibody and streptavidin (0.82% cN1A-specific B cells, Figure 8a), compared to single staining with either the anti-His-tag antibody alone (1.08%, Figure 8b) or streptavidin-BV421 alone (0.85%, Figure 8c).

## 4. Discussion

In this study, we report the successful expression and site-specific biotinylation of recombinant cytosolic 5′-nucleotidase 1A (cN1A) in *Escherichia coli* BL21(DE3)pLysS cells. The resulting biotinylated cN1A retains immunological fidelity and is suitable for autoantibody detection in serological assays and, critically, the isolation of antigen-specific B cells from patient blood samples.

Western blot analyses revealed a clear molecular weight shift between the biotinylated and non-biotinylated forms of cN1A, confirming both the incorporation of the AviTag and successful biotinylation. Crucially, the biotinylated cN1A was robustly recognised by both rabbit and human anti-cN1A antibodies, validating its immunological fidelity. We observed the presence of multimeric aggregates, including putative trimers and higher-order complexes, which were evident in the recombinant cN1A and appeared more pronounced following biotinylation. Although the native structure of cN1A has not yet been elucidated, other cytosolic 5′-nucleotidases, such as cN-II, are known to exist as tetramers under native conditions [19]. In cN-II, multimerisation is thought to be regulated by residues at the C-terminus [19]. If cN1A shares a similar structural arrangement, modifications at the C-terminal end—such as biotinylation—may enhance multimerisation, resulting in aggregates that persist even under the denaturing conditions of SDS-PAGE.

In addition, the detection of a lower molecular weight species retaining the biotin tag suggests the presence of a truncated form of cN1A, likely arising from alternative translation initiation or incomplete transcription. As the biotinylation site is located at the C-terminus, its presence in the truncated product indicates that at least the C-terminal portion of the protein is preserved. However, the precise identity and length of this truncated species cannot be determined from the current assay as the binding epitopes of the polyclonal anti-cN1A antibodies used in the Western blot are undefined.

As demonstrated, dual-labelling of cN1A probes with distinct fluorochromes enables reliable identification of cN1A-specific B cells by flow cytometry. These labelled cells can subsequently be isolated by fluorescence-activated cell sorting and used for multiple downstream applications. For example, sorted B cells may be subjected to single-cell RNA sequencing to profile the gene expression or B cell receptor repertoire to identify and characterise antigen-specific immunoglobulin sequences. Additionally, sorted cells can be cultured in vitro for functional assays, such as cytokine production, proliferation assays, or differentiation into antibody-secreting cells. Recombinant antibodies cloned from these B cells can also be expressed and functionally validated, supporting the development of novel diagnostic tools or therapeutic candidates.

This approach is particularly relevant in autoimmune conditions such as inclusion body myositis (IBM), where cN1A has been established as a key autoantigen [7,8,9]. The ability to study cN1A-specific B cells at high resolution offers new opportunities to dissect the humoral immune response in IBM and other autoantibody-mediated diseases.

In summary, we describe a reproducible platform for generating site-specific biotinylated cN1A, suitable for the use in high-affinity streptavidin-based systems. This approach may be broadly applicable to other proteins, facilitating the development of novel diagnostic tools and biomedical assays.

## Figures and Tables

**Table 1:** List of specific kits and equipment.

Reagent	Manufacturer	Catalog No.
GenePulser Xcell module	Bio-Rad Laboratories, Hercules, CA, USA	AU390060
MicroPulser Electroporation Cuvettes, 0.1 cm gap	Bio-Rad Laboratories, Hercules, CA, USA	1652083
Ni-NTA Fast Start Kit	Qiagen, Hilden, Germany	30600
BCA Protein Assay Kit	Pierce Biotechnology, Rockford, IL, USA	23227
PowerBlotter Semi-dry Transfer System	Invitrogen, Carlsbad, CA, USA	PB0012
Nitrocellulose/Filter Paper Sandwiches	Bio-Rad Laboratories, Hercules, CA, USA	1620213
Centrifugal filter units Amicon Ultra-15	Merck KGaA, Darmstadt, Germany	UFC901024
Lyophilised biotin–protein ligase reaction kit	Avidity, Aurora, CO, USA	BirA500-RT
EasySep Human Pan-B cell enrichment kit	StemCell Technologies, Vancouver, BC, Canada	19554

**Table 2:** List of reagents and chemicals.

Reagent	Manufacturer	Catalog No.
Yeast extract	Oxoid, Basingstoke, Hampshire, UK	LP0021
Tryptone	Oxoid, Basingstoke, Hampshire, UK	LP0021
Lennox LB broth powder	Invitrogen, Carlsbad, CA, USA	12780052
Lennox LB agar	Invitrogen, Carlsbad, CA, USA	22700025
Phosphate Buffered Saline tablets	Thermo Fisher Scientific, Paisley, UK	18912-014
Skimmed milk powder	Merck KGaA, Darmstadt, Germany	115363
Tris Buffered Saline tablets	Sigma-Aldrich, St. Louis, MO, USA	4155256
SureCast Acrylamide 40%	Invitrogen, Carlsbad, CA, USA	HC2040
Tris Base	Sigma-Aldrich, St. Louis, MO, USA	T6066
Glycine	CSA Scientific, Melbourne, VIC, Australia	GA007-500G
Glycerol	Univar Solutions, Downers Grove, IL, USA	1009311
0.04% Bromophenol Blue	Sigma-Aldrich, St. Louis, MO, USA	318744
Sodium Dodecyl Sulphate (SDS)	Sigma-Aldrich, St. Louis, MO, USA	L3771
Ammonium Persulphate (APS)	Pierce Biotechnology, Rockford, IL, USA	17874
TEMED	Pierce Biotechnology, Rockford, IL, USA	17919
Precision Plus Protein Standards	Bio-Rad Laboratories, Hercules, CA, USA	1610374
Coomassie Brilliant Blue R-250	Bio-Rad Laboratories, Hercules, CA, USA	161-0400
Tween-20	Sigma-Aldrich, St. Louis, MO, USA	P2287
Triton X-100	Sigma-Aldrich, St. Louis, MO, USA	X100
Clarity Western ECL substrate	Bio-Rad Laboratories, Hercules, CA, USA	1705060
Bicine	Sigma-Aldrich, St. Louis, MO, USA	B3876
Foetal Calf Serum	Serana, Melbourne, VIC, Australia	S-FBSPG-AU-015
RPMI 1640	Thermo Fisher Scientific, Paisley, UK	11835055
0.5 M EDTA	Life Technologies, Grand Island, NY, USA	15575-020

**Table 3:** Media and buffers.

Component	Final Concentration
**SOB medium ^1^**	
Tryptone	0.02% *w*/*v*
Yeast Extract	0.005% *w*/*v*
Sodium Chloride	8.6 mM
Potassium Chloride	2.5 mM
Magnesium Chloride	10 mM
Magnesium Sulphate	10 mM
**SOC medium ^1^**	
Tryptone	0.02% *w*/*v*
Yeast Extract	0.005% *w*/*v*
Sodium Chloride	8.6 mM
Potassium Chloride	2.5 mM
Magnesium Chloride	10 mM
Magnesium Sulphate	10 mM
Glucose	20 mM
**Cell lysis buffer, pH 8.0**	
Tris base	50 mM
NaCl	150 mM
Triton X-100	0.5% *v*/*v*
**Urea denaturing buffer, pH 8.0**	
Tris base	50 mM
Urea	8 M
**SDS-PAGE running buffer**	
Tris base	25 mM
Glycine	192 mM
SDS ^2^	0.1% *w*/*v*
**2× sample loading buffer**	
Tris-HCl, pH6.8	65.8 mM
SDS ^2^	2% *w*/*v*
Glycerol	26.3% *v*/*v*
Bromophenol blue	0.01% *w*/*v*

^1^ Sterilised and stored at room temperature. Supplemented with 50 µg/mL kanamycin and 34 µg/mL chloramphenicol immediately before use unless stated otherwise. ^2^ Prepared as 20% *w*/*v* stock and stored at ambient temperature.

**Table 4:** Features of BL21(DE3)pLysS *E. coli* strain.

Feature	Genotype	Critical Features
BL21	B F^−^ *ompT gal dcm lon hsdSB* (r^−^ m^−^)	Deficient in Lon and OmpT proteases
λ(DE3)	*lacI* lacUV5-T7p07 ind1 sam7 nin5	T7 RNA polymerase under the control of the lac UV5 promoter
pLysS	T7p20 orip15A(Cm^R^)	Chloramphenicol resistance; T7 phage lysozyme—reduced expression when not induced

**Table 5:** Induction Conditions Tested for the Optimisation of Protein Production.

Test Number	OD_600_	Induction Duration (h)	Induction Temperature (°C)	IPTG ^1^ Concentration (mM)
1	0.6	16	23	0.5
2	1.0	16	23	0.5
3	Uninduced control	16	23	0
4	0.6	16	23	1.0
5	1.0	4	37	0.5
6	1.0	16	23	1.0
7	0.6	4	37	0.5
8	0.6	4	37	1.0
9	Uninduced control	4	37	0
10	1.0	4	37	1.0

^1^ IPTG = Isopropyl β-D-1-thiogalactopyranoside.

**Table 6:** Bovine serum albumin standard dilution scheme for the BCA microplate procedure.

Vial	Volume of Diluent (µL)	Volume and Source of BSA (µL)	Final BSA Concentration (µg/mL)
A	0	60 of stock	2000
B	25	75 of stock	1500
C	65	65 of stock	1000
D	35	35 of vial B dilution	750
E	65	65 of vial C dilution	500
F	65	65 of vial E dilution	250
G	65	65 of vial F dilution	125
H	80	20 of vial G dilution	25
I	80	0	0 (Blank)

**Table 7:** List of antibody pairs used for Western blotting.

Primary Ab	Dilution	Manufacturer	Secondary Ab	Dilution	Manufacturer
Human α-human cN1A	1:1000	In-house	Goat α-human IgG/IgM/IgA—HRP	1:10,000	Invitrogen
Rabbit α-human cN1A	1:500	Sigma-Aldrich	Goat α-rabbit IgG—HRP	1:10,000	Invitrogen
-	-	-	Streptavidin-HRP	1:10,000	BioLegend

**Table 8:** Fluorochrome-conjugated antibodies for flow cytometry staining.

Antibody Conjugate	Manufacturer	Catalogue #	Volume (μL) per 1 × $10^{6}$ Cells
CD19-APC-Fire750	BioLegend, San Diego, CA, USA	302257	5
Streptavidin-BV421	BioLegend, San Diego, CA, USA	405226	2.5
Polyhis-tag-CF488	Merck KGaA, Darmstadt, Germany	SAB4600048	5
eFluor 506 viability dye	Invitrogen, Carlsbad, CA, USA	65-0866-14	2.5

## Data Availability

Raw gel and blot images can be accessed via DOI: 10.6084/m9.figshare. 28878911.

## Supplementary Materials

The following supporting information can be downloaded at https://www.mdpi.com/article/10.3390/mps8030066/s1. *E. coli*-optimised DNA sequence of human cytosolic 5′-nucleotidase 1A and the translated protein sequence; Figure S1: Preferred codon usage table for *E. coli*. Printed from SnapGene Viewer v 7.0.

## Abbreviations

The following abbreviations are used in this manuscript:

cN1A	Cytosolic 5′-nucleotidase 1A
5NT	5′-nucleotidase
IBM	Inclusion Body Myositis
*E. coli*	Escherichia coli
IPTG	Isopropyl β-D-1-thiogalactopyranoside
EDTA	Ethylenediaminetetraacetic acid
FCS	Foetal Calf Serum
SDS-PAGE	Sodium Dodecyl Sulphate–Polyacrylamide Gel Electrophoresis
BCA	Bicinchoninic Acid Assay
