# Supplementary material for: Expression and Site-Specific Biotinylation of Human Cytosolic 5′-Nucleotidase 1A in Escherichia coli
Source: Methods Protoc. 2025 Jun 18;8(3):66. doi: 10.3390/mps8030066 (PMC12195718; doi:10.3390/mps8030066)
Supplement: Supplementary file 1 [file mps-08-00066-s001.zip › mps-3636047-supplementary.pdf]

Supplementary Data 1

E. coli optimised DNA sequence of human cytosolic 5′- nucleotidase 1A

```
CATATGCACCACCACCACCACCACATGGAACCCGGACAACCAAGGGAACCCCAGGAACCGCGTGAACC
GGGTCCGGGCGCGGAAACCGCGGCGGCGCCGGTTTGGGAGGAAGCGAAAATCTTCTACGATAACCTGG
CGCCGAAGAAAAAGCCGAAAAGCCCGAAGCCGCAGAACGCGGTTACCATCGCGGTGAGCAGCCGTGCG
CTGTTTCGTATGGACGAGGAACAGCAAATTTATACCGAGCAGGGTGTTGAGGAATACGTGCGTTATCA
ACTGGAGCATGAAAACGAGCCGTTCAGCCCGGGTCCGGCGTTCCCGTTTGTTAAGGCGCTGGAAGCGG
TGAACCGTCGTCTGCGTGAGCTGTACCCGGACAGCGAAGATGTTTTTGACATTGTGCTGATGACCAAC
AACCACGCGCAAGTTGGTGTGCGTCTGATCAACAGCATTAACCACTACGACCTGTTCATCGAGCGTTT
TTGCATGACCGGTGGFAACAGCCCGATTTGCTACCTGAAAGCGTATCACACCAACCTGTATCTGAGCG
CGGATGCGGAAAAGGTTCGTGAGGCGATCGACGAAGGCATTGCGGCGGCGACCATCTTCAGCCCGAGC
CGTGACGTGGTTGTGAGCCAGAGCCAACTGCGTGTTGCGTTCGACGGTGATGCGGTGCTGTTTAGCGA
TGAAAGCGAGCGTATTGTGAAAGCGCACGGCCTGGACCGTTTCTTTGAACACGAGAAAGCGCACGAGA
ACAAGCCGCTGGCGCAGGGTCCGCTGAAAGGCTTCCTGGAAGCGCTGGGTCGTCTGCAGAAGAAGTTT
TACAGCAAGGGCCTGCGTCTGGAATGCCCGATCCGTACCTATCTGGTTACCGCGCGTAGCGCGGCGAG
CAGCGGTGCGCGTGCGCTGAAGACCCTGCGTAGCTGGGGCCTGGAAACCGATGAGGCGCTGTTCCTGG
CGGGTGCGCCCAAGGGTCCGCTGCTGGAGAAGATCCGTCCGCACATTTTCTTTGACGATCAGATGTTT
CACGTGGCGGGTGCGCAAGAAATGGGCACCGTTGCGGCGCACGTGCCGTATGGTGTGGCGCAGACCCC
GCGTCGTACCGCGCCGGCGAAGCAAGCGCCGAGCGCGGCTT
```

Translated amino acid sequence

```
HMHHHHHHMEPGQPREPQEPREPGPGAETAAAPVWEEAKIFYDNLAPKKKPKSPKPQNAVTIAVSSRA
LFRMDEEQQIYTEQGVEEYVRYQLEHENEPFSPGPAFPFVKALEAVNRRLRELYPDSEDVFDIVLMTN
NHAQVGVRLINSINHYDLFIERFCMTGXNSPICYLKAYHTNLYLSADAEKVREAIDEGIAAATIFSPS
RDVVVSQSQLRVAFDGDAVLFSDESERIVKAHGLDRFFEHEKAHENKPLAQGPLKGFLEALGRLQKKF
YSKGLRLECPIRTYLVTARSAASSGARALKTLRSWGLETDEALFLAGAPKGPLLEKIRPHIFFDDQMF
HVAGAQEMGTVAAHVPYGVAQTPRRTAPAKQAPSAA
```

Codon Usage Table: Escherichia coli
Green = preferred codon

Second Position

First Position	U	C	A	G	Third Position
U	UUU → Phe (F) **0.58**	UCU → Ser (S) **0.14**	UAU → Tyr (Y) **0.57**	UGU → Cys (C) **0.45**	U
U	UUC → Phe (F) **0.42**	UCC → Ser (S) **0.15**	UAC → Tyr (Y) **0.43**	UGC → Cys (C) **0.55**	C
U	UUA → Leu (L) **0.13**	UCA → Ser (S) **0.14**	UAA → * **0.59**	UGA → * **0.33**	A
U	UUG → Leu (L) **0.13**	UCG → Ser (S) **0.15**	UAG → * **0.08**	UGG → Trp (W) **1.00**	G
C	CUU → Leu (L) **0.11**	CCU → Pro (P) **0.17**	CAU → His (H) **0.58**	CGU → Arg (R) **0.36**	U
C	CUC → Leu (L) **0.10**	CCC → Pro (P) **0.13**	CAC → His (H) **0.42**	CGC → Arg (R) **0.37**	C
C	CUA → Leu (L) **0.04**	CCA → Pro (P) **0.19**	CAA → Gln (Q) **0.33**	CGA → Arg (R) **0.07**	A
C	CUG → Leu (L) **0.49**	CCG → Pro (P) **0.51**	CAG → Gln (Q) **0.67**	CGG → Arg (R) **0.11**	G
A	AUU → Ile (I) **0.50**	ACU → Thr (T) **0.17**	AAU → Asn (N) **0.47**	AGU → Ser (S) **0.16**	U
A	AUC → Ile (I) **0.40**	ACC → Thr (T) **0.41**	AAC → Asn (N) **0.53**	AGC → Ser (S) **0.27**	C
A	AUA → Ile (I) **0.09**	ACA → Thr (T) **0.15**	AAA → Lys (K) **0.76**	AGA → Arg (R) **0.05**	A
A	AUG → Met (M) **1.00**	ACG → Thr (T) **0.27**	AAG → Lys (K) **0.24**	AGG → Arg (R) **0.03**	G
G	GUU → Val (V) **0.26**	GCU → Ala (A) **0.16**	GAU → Asp (D) **0.63**	GGU → Gly (G) **0.33**	U
G	GUC → Val (V) **0.21**	GCC → Ala (A) **0.27**	GAC → Asp (D) **0.37**	GGC → Gly (G) **0.39**	C
G	GUA → Val (V) **0.16**	GCA → Ala (A) **0.22**	GAA → Glu (E) **0.68**	GGA → Gly (G) **0.12**	A
G	GUG → Val (V) **0.37**	GCG → Ala (A) **0.35**	GAG → Glu (E) **0.32**	GGG → Gly (G) **0.16**	G

Figure S1: Preferred codon usage table for *E. coli*. Printed from SnapGene Viewer v 7.0
